# The right ventricular end-systolic volume index predicts mortality in patients with cardiac amyloidosis

**DOI:** 10.1186/1532-429X-18-S1-P139

**Published:** 2016-01-27

**Authors:** Ke Wan, Yong Luo, Dan Yang, Hong Liu, Yucheng Chen

**Affiliations:** grid.13291.380000000108071581Sichuan University, Chengdu, China

## Background

Cardiac amyloidosis (CA) is generally characterized by a poor prognosis and significantly increased mortality. Although right ventricular (RV) dysfunction is strong predictor of outcome in AL amyloidosis, there are limited data regarding the prognostic significance of RV size. We sought to investigate whether cardiovascular magnetic resonance (CMR) assessment of RV size has prognostic value in CA.

## Methods

Consecutive patients referred for CMR for possible CA were retrospectively evaluated. Diagnosis of CA was based on that all patients had positive Congo red staining of biopsy (periumbilical fat, rectum, kidney), and the presence of a typical pattern of subendocardial late gadolinium enhancement. The patients underwent CMR with measurement of left ventricular (LV) and RV volumes, mass and ejection fraction.

## Results

69 patients (41 males (59.4%), age 60 ± 11 years) were included, CA was diagnosed in 47 (68.1%). Age, gender, New York Heart Association class, medications, serological biomarkers, and electrocardiographic voltage were recorded. The parameters measured by MRI were also recorded, such as the left ventricular end-diastolic volume (LVEDV), the right ventricular end-diastolic volume (RVEDV), the left ventricular mass (LV mass). Mean age was 61 ± 10 years with 28 men (59.5%) in CA. New York Heart Association class >II was noted in 24 patients (51.1%) and low voltage on electrocardiogram (QRS amplitude of ≤0.5 mV in all limb leads or ≤1 mV in all precordial leads) in 23(48.9%). At a median follow-up of 21 months, there were 27 deaths (55.3%). On univariate Cox regression analysis, New York Heart Association class>II, the left ventricular mass index (LV massi), the right ventricular ejection fraction (RVEF), the right ventricular end-systolic volume index (RVESVI) were associated with increased mortality. On multivariable Cox proportional analysis, only RVESVI was significantly associated with increased mortality (hazard ratio1.037, 1.001 to 1.075, p = 0.043). RVESVI had the better reliable diagnostic power for survival compared with NYHA class by ROC curves, with an area under the curve equal to 0.738.The receiver-operator characteristic curve-derived cut-off value of RVESVI of 32 mL/m^2^ predicted mortality, (x^2^: 10.084, P < 0.001).

## Conclusions

Our data show that in patients with Cardiac Amyloidosis, the right ventricular end-systolic volume index is a powerful, independent predictor of longer-term survival. Cardiovascular magnetic resonance assessment of RV size is important in the evaluation and risk stratification of in these patients.

